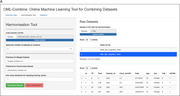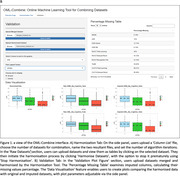# OML‐Combine: Online Machine Learning tool for Combining datasets to increase power and facilitate multi‐centre collaboration on Alzheimer's disease studies

**DOI:** 10.1002/alz.085143

**Published:** 2025-01-09

**Authors:** Chris Dalas Christodoulides, Rodrigo Canovas, Vincent Dore, James D. Doecke, Simon M Laws, Michael W Weiner, Christopher C. Rowe, Victor L Villemagne, Colin L. Masters, Jurgen Fripp, Paul Maruff, Rosita Shishegar

**Affiliations:** ^1^ The Australian e‐Health Research Centre, CSIRO, Melbourne, VIC Australia; ^2^ Australian E‐Health Research Centre, CSIRO, Melbourne, VIC Australia; ^3^ CSIRO Health and Biosecurity, Australian E‐Health Research Centre, Brisbane, QLD Australia; ^4^ Department of Molecular Imaging, Austin Health, Melbourne, VIC Australia; ^5^ The Australian e‐Health Research Centre, CSIRO, Brisbane, QLD Australia; ^6^ Centre for Precision Health, Edith Cowan University, Joondalup, Western Australia Australia; ^7^ San Francisco Veterans Administration Medical Center (SFVAMC), San Francisco, CA, CA USA; ^8^ Molecular Research and Therapy, Austin Health and University of Melbourne, Heidelberg, VIC Australia; ^9^ The Florey Institute of Neuroscience and Mental Health, The University of Melbourne, Parkville, VIC Australia; ^10^ Department of Molecular Imaging & Therapy, Austin Health, Heidelberg, VIC Australia; ^11^ University of Pittsburgh, Pittsburgh, PA USA; ^12^ Florey Institute of Neuroscience and Mental Health, Parkville, VIC Australia; ^13^ The Australian e‐Health Research Centre, Commonwealth Scientific and Industrial Research Organisation, Brisbane, QLD Australia; ^14^ Cogstate Ltd., Melbourne, VIC Australia; ^15^ Turner Institute for Brain and Mental Health, Monash University, Melbourne, VIC Australia; ^16^ School of Psychological Sciences and Turner Institute for Brain and Mental Health, Monash University, Monash, VIC Australia; ^17^ Austin Health, Heidelberg, VIC Australia; ^18^ The Australian e‐Health Research Centre, CSIRO, Parkville, VIC Australia

## Abstract

**Background:**

Diagnostic and prognostic decisions about Alzheimer’s disease (AD) are more accurate when based on large data sets. We developed and validated a machine learning (ML) data harmonization tool for aggregation of prospective data from neuropsychological tests applied to study AD. The online ML‐combine application (OML‐combine app) allows researchers to utilize the ML‐harmonization method for harmonization of their own data with that from other large available data bases (e.g. AIBL) to enable development of their own neuropsychological models of AD.

**Method:**

The OML‐Combine application implements an established neuropsychological test data harmonization method^1^ based on non‐parametric multivariate imputation using random forests (missForest)^2^. Test data not included in a cohort is classified as missing and imputed using known data from the cohort based on information known from other studies^1^. A web‐based R‐Shiny application was developed to facilitate harmonization of data from different cohorts and visualise outcomes. OML‐combine also calculates percentages of missing values for each test score across the pooled dataset allowing decisions about the validity of harmonized data. OML‐combine also allows harmonization of multiple datasets simultaneously.

**Result:**

The R Shiny package was used to produce an interactive data harmonization tool. Figure 1 displays the interface, showing results from an example harmonization and validation step using simulated data from AIBL (N=1813) and ADNI (N=1945). In the validation tab, users are provided with a figure of the distributions of both raw and harmonized datasets, including predicted test scores and an accuracy measurement for each score. These can be used to validate the outcomes and compare them to known relationships established from the raw data for each dataset.

**Conclusion:**

OML‐Combine facilitates the harmonization of neuropsychological test data from established AD cohort studies. Visualization of predicted test scores and the original data sets can thereby assist with decisions about accuracy of harmonized data and can provide a basis for adjustment of inputs to optimize models. This allows researchers to combine their own data with that from other currently available studies to improve diagnostic and prognostic models of AD.

References:

^1^doi: 10.1002/alz.044302

^2^doi:10.1093/bioinformatics/btr597